# Editorial: Stress and the brain: advances in neurophysiological measures for mental stress detection and reduction

**DOI:** 10.3389/fnrgo.2024.1466783

**Published:** 2024-08-14

**Authors:** Stefania Coelli, Eleonora Maggioni, Martin O. Mendez

**Affiliations:** Department of Electronics, Information and Bioengineering (DEIB), Politecnico di Milano, Milano, Italy

**Keywords:** mental stress, emotion, autonomic nervous system monitoring, multimodal analysis, affective computing, cognitive performance, neurophysiological monitoring

Stress is a multidisciplinary field of research that brings together psychology, medicine (ranging from physiology to cardiology and psychiatry), sociology, and bioengineering. Recent efforts have been made to improve our understanding of the effects of stress on physical and mental health and wellbeing, and to provide solutions for preventing and better managing conditions at risk for pathology. Indeed, stress is the disease of our times, affecting people regardless of gender, age, or social status. It is associated with several pathological conditions, thus, making advances in reliable monitoring, measurement, and assessment of mental states is critical for early intervention and prevention of chronic stress.

Stress research has advanced significantly over the years, but challenges remain in accurately identifying, measuring, and mitigating stress in real-world settings. Technological innovations and methodological improvements have opened new avenues for exploring how stress affects the brain and the body. Our Research Topic aims to highlight recent advances and interdisciplinary efforts in the field of stress research.

This Research Topic brings together a collection of articles that exemplify the multidisciplinary approach needed to thoroughly address the complex nature of stress. Contributions range from the theoretical conceptualization of stress as a multimodal domain through the measurement and classification of the different domains of acute stress, to the characterization of the health effects of chronic stress due to work conditions.

## Preview of the Research Topic

This Research Topic features four contributions: one Hypothesis and Theory article and three research articles.

The Hypothesis and Theory article by Steffen, proposes the Research Domain Criteria (RDoC) as an integrative framework for stress research and gives a complete and extremely clear overview of the current advancements and future needs in the field. Specifically, RDoC is not only described as a multi-modal research approach based on different psychophysiological domains (i.e., cognitive, valence, arousal, sensorimotor and social), but also as a promising framework for integrating psychotherapy and stress management interventions, such as biofeedback and mindfulness. Therefore, this insightful contribution foresees the RDoC as the basis for conceptualizing future research on mental stress integrating information from physiological data (e.g., heart rate variability (HRV) and respiration) and molecular data (cortisol) to assess and improve chronic stress management.

The usefulness of a multimodal approach is also supported by both the original research articles contributed by Tervonen et al. and Bruin et al., which consider different physiological parameters. Specifically, Tervonen et al. extracted parameters from electrocardiography (ECG), electroencephalography (EEG), electrodermal activity (EDA) and eye movements data to construct models able to classify different types of acute stress. In fact, the Maastricht Acute Stress Test (MAST) was used to manipulate the emotional response of volunteers using two types of stressors:

- physiological stress through physical stimuli (cold pressure)
- psychosocial stress through mathematical calculation and evaluation.

More than 100 features were extracted from the recorded signals during the MAST protocol, testing different window lengths, and several models were trained in changing classifiers and feature sets.

Interestingly, using an explainable artificial intelligence approach, this article highlighted the importance of the different physiological parameters to characterize the specific stressors, demonstrating that it is possible to discern stress types.

On the other hand, Bruin et al. used a combination of features derived from EDA, ECG and facial expression data to classify volunteers' emotional responses to stressors into two levels of arousal and valence for each kind of stress task. In line with the previously described work, stressors were of different domains (i.e., cognitive, social and emotional). Also in this case, more than 100 features were extracted from the acquired data and different models were trained using subsets of features. Results showed that, basing the ground truth on self-rated arousal and valence, arousal seems to be better captured by physiological parameters, while valence by features extracted from facial expressions (video) data.

While these two contributions focused on the detection and classification of acute stress responses, in terms of emotional reactions to stressful stimuli, the article by Delgado-Aranda et al. explored the effects of a chronic stressful work condition on the cardiovascular system. Specifically, sleep-wake cycle disruption caused by shift work in women was studied trough the analysis of HRV complex pattern (multifractality), allowing the exploration of the regulatory mechanisms of the autonomic nervous system (ANS) during sleep. Their results suggest an abnormal regulation in shift workers that could be associated with cardiovascular stress.

## Final remarks

The collected articles provide an insightful overview of current advances in neurophysiological approaches to comprehensively explore the effects of stress conditions in everyday life. However, measuring and classifying types of stress based on physiological responses is only the beginning of a long road. Progress in stress management will only be achieved if efforts are made to enhance social awareness, to concretely prevent stressful conditions, and to improve coping strategies.

This Research Topic underscores the critical importance of a multidisciplinary approach in addressing the multifaceted nature of stress. By combining theoretical frameworks, advanced measurement techniques, and practical interventions, researchers can pave the way for more effective stress management solutions. The knowledge gained from these studies not only advances our scientific understanding, but also has the potential to significantly impact public health strategies and individual wellbeing.

## Author contributions

SC: Writing – review & editing, Writing – original draft. EM: Writing – review & editing, Writing – original draft. MM: Writing – review & editing, Writing – original draft.

